# Duplex recombinase aided amplification-lateral flow dipstick assay for rapid distinction of *Mycobacterium tuberculosis* and *Mycobacterium avium complex*

**DOI:** 10.3389/fcimb.2024.1454096

**Published:** 2024-10-10

**Authors:** Ke Chen, Junze Zhang, Simeng Wang, Zhengjun Yi, Yurong Fu

**Affiliations:** ^1^ Department of Medical Microbiology, School of Basic Medicine, Shandong Second Medical University, Weifang, Shandong, China; ^2^ Clinical Laboratory, Weifang Second People’s hospital, Weifang, Shandong, China; ^3^ School of Medical Laboratory, Shandong Second Medical University, Weifang, Shandong, China

**Keywords:** *Mycobacterium tuberculosis* (MTB), *Mycobacterium avium complex* (MAC), recombinase-aided amplification (RAA), lateral flow dipstick (LFD), rapid distinction

## Abstract

**Objectives:**

This study aims to develop a novel diagnostic approach using the recombinase aided amplification-lateral flow dipstick(RAA-LFD) assay for the distinction of *Mycobacterium tuberculosis* (MTB) and *Mycobacterium avium complex* (MAC), enabling rapid and convenient as well as accurate identification of them in clinical samples

**Methods:**

Our study established a duplex RAA-LFD assay capable of discriminating between MTB and MAC. Based on the principles of RAA primer and probe design, specific primers and probes were developed targeting the MTB *IS6110* and the MAC *DT1* separately. Optimization of reaction time points and temperatures was conducted, followed by an evaluation of specificity, sensitivity, and reproducibility. The established detection method was then applied to clinical samples and compared with smear microscopy, liquid culture, LAMP, and Xpert/MTB RIF in terms of diagnostic performance

**Results:**

The complete workflow allows for the effective amplification of the MTB *IS6110* and MAC *DT1* target sequences at constant 37°C within 20min, and the amplification products can be visually observed on the LFD test strip. This method exhibits high specificity, showing no cross-reactivity with nucleic acids from *M. kansassi*, *M. abscessus, M. gordonae, M. chelonae, M. fortuitum, M. scrofulaceum, M. malmoense, M. chimaera, M. szulgai* and common respiratory pathogens. It also demonstrates high sensitivity, with a detection limit as low as 10^2^ CFU/mL. Additionally, the method’s Coefficient of Variation (CV) is less than 5%, ensuring excellent repeatability and reliability. Furthermore, clinical performance evaluations, using Xpert/MTB RIF as the gold standard, demonstrated that the duplex RAA-LFD assay achieves a sensitivity of 92.86% and a specificity of 93.75%. It is also noteworthy that the assay exhibits considerable diagnostic efficacy in smear-negative patients

**Conclusions:**

Our study introduces a rapid, specific, and sensitive duplex RAA-LFD assay for the discriminatory diagnosis of MTB and MAC. This method represents a significant advancement in the field of infectious disease diagnostics, offering a valuable tool for rapid detection and management of MTB and MAC infections. The implementation of this approach in point-of-care settings could greatly enhance TB control and prevention efforts, especially in resource-limited environments.

## 1 Introduction

Tuberculosis, caused by MTB, kills 1.5 million people every year and has tremendous negative health and economic effects, especially in underdeveloped countries (Bagcchi, 2023). *Non-tuberculous Mycobacterium* (NTM) refers to strains of the genus *Mycobacterium* other than MTB complex group and *Mycobacterium leprae*. Over 90% of clinical NTM isolates belongs to *Mycobacterium intracellular complex* (MAC), *Mycobacterium kansasii*, or *Mycobacterium abscessus* (Tan et al., 2021).The accurate and timely diagnosis of TB and its differentiation from diseases caused by *non-tuberculous mycobacteria* (NTM) is crucial for appropriate treatment and control measures (Gopalaswamy et al., 2020; Thomson and Yew, 2009). Traditional diagnostic methods, including sputum smear microscopy, culture, and nucleic acid amplification tests (NAATs), have limitations in terms of sensitivity, specificity, turnaround time, and operational complexity. Therefore, innovative diagnostic methods need to be developed (Hermes et al., 2018).

In recent years, isothermal amplification techniques have emerged as promising tools for nucleic acid detection, offering simplicity, rapidity, and high efficiency at a constant temperature (Silva Zatti et al., 2020; Wei et al., 2023). Among these, the recombinase aided amplification (RAA) has shown potential for point-of-care testing (POCT) diagnostics due to its low operational complexity and ability to produce results within minutes at a single temperature (Zhao et al., 2021). RAA’s utility in detecting a wide range of pathogens has been documented (Wang et al., 2021). However, its application, in differentiating TB from MAC infections, remains underexplored.

The objective of this study is to develop and evaluate a duplex RAA-LFD assay for the discriminatory diagnosis of MTB and MAC. This approach aims to address the limitations of current diagnostic methods by providing a rapid, specific, and sensitive tool for TB diagnosis and differentiation from MAC infection, which is essential for the accurate treatment and control of *mycobacterial* diseases.

## 2 Materials and methods

### 2.1 Primers and probe design for duplex RAA-LFD

We selected the conserved and specific MTB *IS6110* and the MAC *DT1* (Gene ID: L0454) as the target markers. Primers and probes were designed within the conserved regions by using Oligo 7 software, according to the principles of RAA primer and probe design. All primers and probes were synthesized by Sangon Biotech Co., Ltd (Shanghai, China) using high-performance liquid chromatography (HPLC). Details of the final primers and probes are shown in **Table 1**.

**Table 1:** The primers and probes used in MTB and MAC duplex RAA-LFD assay.

Gene Primers/probes	Sequences(5’-3’)	Size
IS6110_—_Forward	5’-CCCCATCGACCTACTACGACCACATCAACCGGGAG-3’	
IS6110_—_Reverse	5’-Biotin-TCACGGTTCAGGGTTAGCCACACTTTGCGGGCAC-3’	148 bp
IS6110_—_Probe	5’-FITC-CTCAAGGAGCACATCAGCCGCGTCCACGCC-THF-CCAACTACGGTGTTTA-C3 Spacer-3’	
DT1_—_Forward	5’- CTTTCACCTGCTCCATTCCCGTTCTTCACACC-3’	
DT1_—_Reverse	5’-TAMRA-GACGACGGCGTTCGAAATGGCACACATCAGC-3’	231 bp
DT1_—_Probe	5’-Digxin-TGTCCGACCGTGTTGCGCTCGTCGTAGCT-THF-TCCAGGCCGATCCAT-C3 Spacer -3’	

### 2.2 Duplex RAA-LFD assay

The RAA assays were performed using multienzyme isothermal rapid amplification nfo kit (DNA) purchased from Qitian Biotech Co., Ltd (Jiangsu, China). The RAA system (50μL per reaction) contained 25μL of buffer V, 0.3μL of IS6110-Forward, 0.3μL of IS6110-Reverse, 0.6μL of IS6110-Probe, 0.7μL of DT1-Forward, 0.7μL of DT1-Reverse,0.6μL of DT1-Probe,4μL of template, 2.5μL of magnesium acetate and 15.3μL of nuclease-free water. We spined down and repeat three times to ensure thorough mixing. After a brief centrifuge, we transfered the tubes to a metal bath and incubate at 37°C for 20min. Negative control with nuclease-free water was included in each run. The amplification products were analyzed by using lateral flow dipstick (LFD) purchased from Tiosbio Co., Ltd (Beijing China). After heating, we transfered 2μL of the amplification product into a 1.5mL EP tube containing 48μL of RNase-free water solution, then mix well and insert the colloidal gold strip’s sample end into the tube. The changed were observed on the colloidal gold test strip detection line.

### 2.3 Specificity of RAA assay

For specificity analysis, the assay was evaluated with *M.kansassi*, *M.abscessus*, *M.gordonae*, *M.chelonae*, *M.fortuitum*, *M.scrofulaceum*, *M.malmoense*, *M.chimaera*, *M.szulgai*,*Klebsiella pneumoniae*, *Pseudomonas aeruginosa*, *Streptococcus pneumoniae*, *Hemophilus influenzae*, *Acinetobacter baumannii*, *Staphylococcus aureus*, *Stenotrophomonas maltophilia* to identify the specificity of RAA-LFD assay in the diagnosis of MTB and MAC. The DNA template was extracted by the CTAB method. The DNA template from MTB and MAC, and nuclease-free water were used as a positive and blank control, respectively.

### 2.4 Sensitivity analysis

The fresh cultures of MTB and MAC strains were separately resuspended to a turbidity equivalent of 1mg/mL. The suspension was then diluted in a 10-fold gradient using physiological saline, with each dilution (1mL) tested using the RAA-LFD assay. Both MTB and MAC are slow-growing *mycobacteria*, and their culture times are similar. For each dilution level, 100μL of suspension was inoculated onto Löwenstein-Jensen (LJ) medium and incubated at 37°C for 2-4w to record the final colony count. The sensitivity of the method was determined by the average colony count on the LJ medium corresponding to the lowest dilution detected by the duplex RAA-LFD assay. All pathogen experiments were conducted in the TB screening laboratory at Weifang NO.2 People’s Hospital, which complies with Biosafety Level 2 (BSL-2) standard.

### 2.5 Repeatability analysis

The inter-batch assays for duplex RAA-LFD were evaluated by testing two different concentrations: medium concentration (MTB:6.87×10^4^fg/μL,MAC:5.94×10^4^fg/μL), and low concentration (MTB:6.87×10^3^fg/μL,MAC:5.94×10^3^fg/μL).The inter-batch assay reproducibility was asssessed across three independent experiments. The gray level of the detection line was analyzed by ImageJ. Coefficient of Variation (CV%)=(Standard Deviation​/Mean)×100%.

### 2.6 Duplex assay performance for *Mycobacterial* infection

From January to December 2022, 60 patients with suspected TB symptoms were selected as research subjects at Weifang NO.2 People’s hospital. This study protocol was approved by the Ethical Committee of Shandong Second Medical University(2021YX104). Informed consent was obtained from all participants. The specimen types, including bronchoalveolar lavage fluid (BALF) and sputum, were used in the study. The sputum or BALF was liquefied with 4% NaOH, and 1mL of this solution was inactivated at 80°C for 30min. The established duplex RAA-LFD assay, liquid culture, loop-mediated isothermal amplification (LAMP), and Xpert assays were used to test 60 suspected mycobacterium infection samples, with ddH_2_O serving as the negative control and Clinical diagnosis as the positive control to compare the concordance of the detection methods. Based on the diagnostic criteria for TB, the subjects were divided into *mycobacterium* positive and negative group, and The details of clinical specimens used have been presented in the **Supplementary Material**.

### 2.7 Statistical analysis

Statistical analysis was performed using SPSS 21. Clinical data for the included cases were recorded in an Excel spreadsheet. Sensitivity, specificity, positive predictive value, negative predictive value, and accuracy were calculated using Xpert/MTB RIF and clinical diagnosis as reference standards. Detection rates between different methods were compared using McNemar’s test, with a significance level set at α = 0.05. Consistency was evaluated using the Kappa statistic, where a Kappa value ≥0.75 indicates excellent consistency, 0.40 < Kappa < 0.75 indicates moderate consistency, and Kappa < 0.40 indicates poor consistency. Statistical significance was defined as a *p* < 0.05.

## 3 Results

### 3.1 System construction and reaction condition optimization

**Figure 1:**
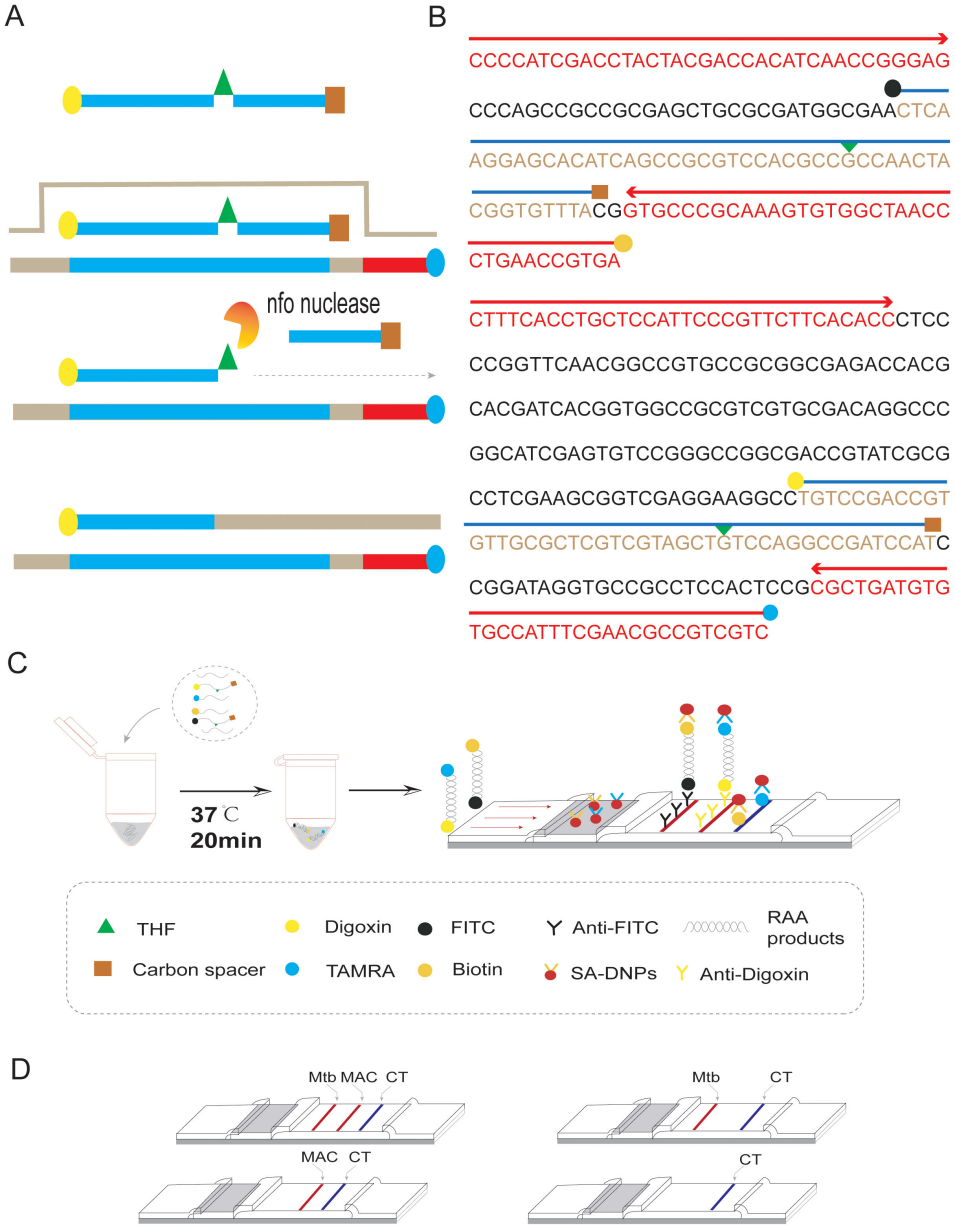
Duplex RAA-LFD differential diagnosis of MTB and MAC experimental procedures **(A)** RAA amplification process: The processed probe and the opposing primer will generate double- stranded amplification products that co-join the two antigenic labels. **(B)** The conserved and specific regions of MTB IS6110 and MAC DT1 gene were selected to design the probes and primers. The differentiation diagnosis is achieved through antigenic modification of two sets of primer-probe combinations. For the MTB assay, the 5’end of the downstream primer is modified with biotin, while the 5’end of the probe is modified with FITC. Both are further modified at the base 31 positions from the 5’end with tetrahydrofuran (THF), and a polymerase extension blocking group, C3 spacer, is attached at the 3’end. For the MAC assay, the 5’end of the downstream primer is modified with rhodamine (TAMRA), and the 5’end of the probe is modified with Digoxin. Additionally, for DT1-R, the bases at positions 30 from the 5’end, respectively, are modified with tetrahydrofuran (THF), and a polymerase extension blocking group, C3 spacer, is present at the 3’end. **(C)** As shown, two groups of probe groups with specific primers were added into the same system. After adding the target nucleotide sequence, the two groups of modified target sequences were obtained after constant temperature amplification at 37°C for 20min (Biotin-S6110-FITC, TAMRA-DT1-Digoxin). After dilution, RAA products combine with SA-DNPs through lateral flow dipstick, and show red detection lines when flowing through the coated Anti-FITC and Anti-Digoxin detection lines, respectively. The quality control lines are coated with TAMRA and biotin. **(D)** Interpretation of the RAA-LFD results. I, a positive result for IS6110 and DT1 (Test line 1, Test line 2 and Control line appear on the LFD); II, a positive result for MTB IS6110 (Test line 1 and Control line appear on the detection region); III, a positive result for MAC DT1 (Test line 2 and Control line appear on the detection region); IV, negative (only the control line appears on the LFD). Note, Test line 1 (Positive result for IS6110) can be judged as positive for MTB. Test line 2(Positive for DTI) can be judged as positive for MAC. Test line 1 and Test line 2 can be judged as both of mixed infection.

**Figure 2:**
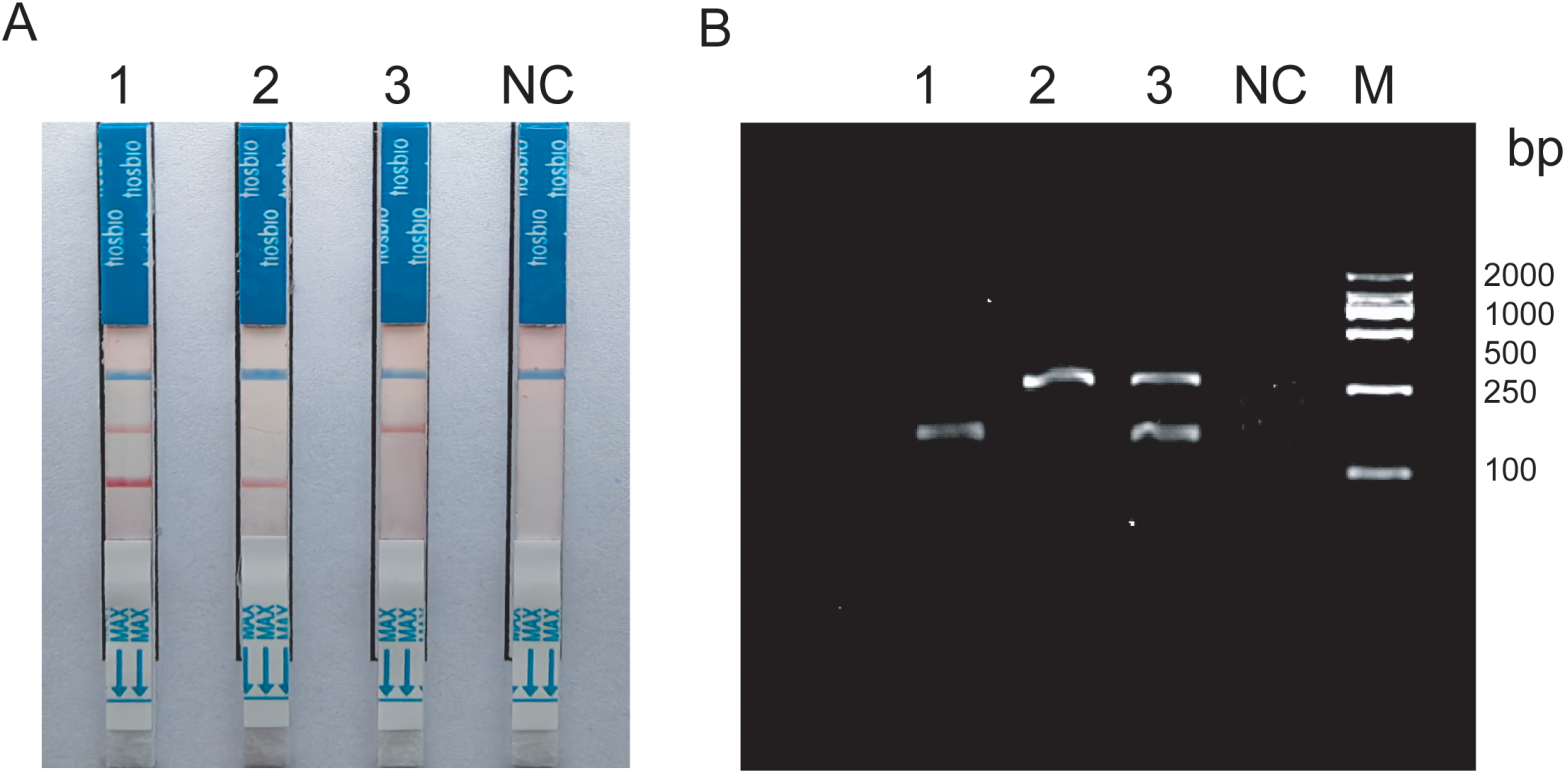
**(A)** Construction of duplex RAA-LFD 1:Mixed template; 2-3:Target bacteria single template; **(B)** Construction of duplex RAA 1-2:Target bacteria single template; 3:Mixed template; NC:ddH_2_O; M:Marker.

The duplex RAA reaction system contains two sets of specificity primers, two types of target pathogen DNA templates and one type of target pathogen DNA template (**Figure 1**). The amplification results were analyzed using LFD assays and agarose gel electrophoresis to verify the specificity between the two targeted strains. The experimental results showed that in the same detection system, a red line on the dipstick was only observed when the corresponding target pathogen DNA template was added (**Figure 2**). The duplex RAA agarose detection results were basically consistent with the LFD, proving good specificity between the two targeted strains without non-specific amplification or cross-reactivity. Thus, the system effectively differentiates and diagnoses MTB and MAC.

**Figure 3:**
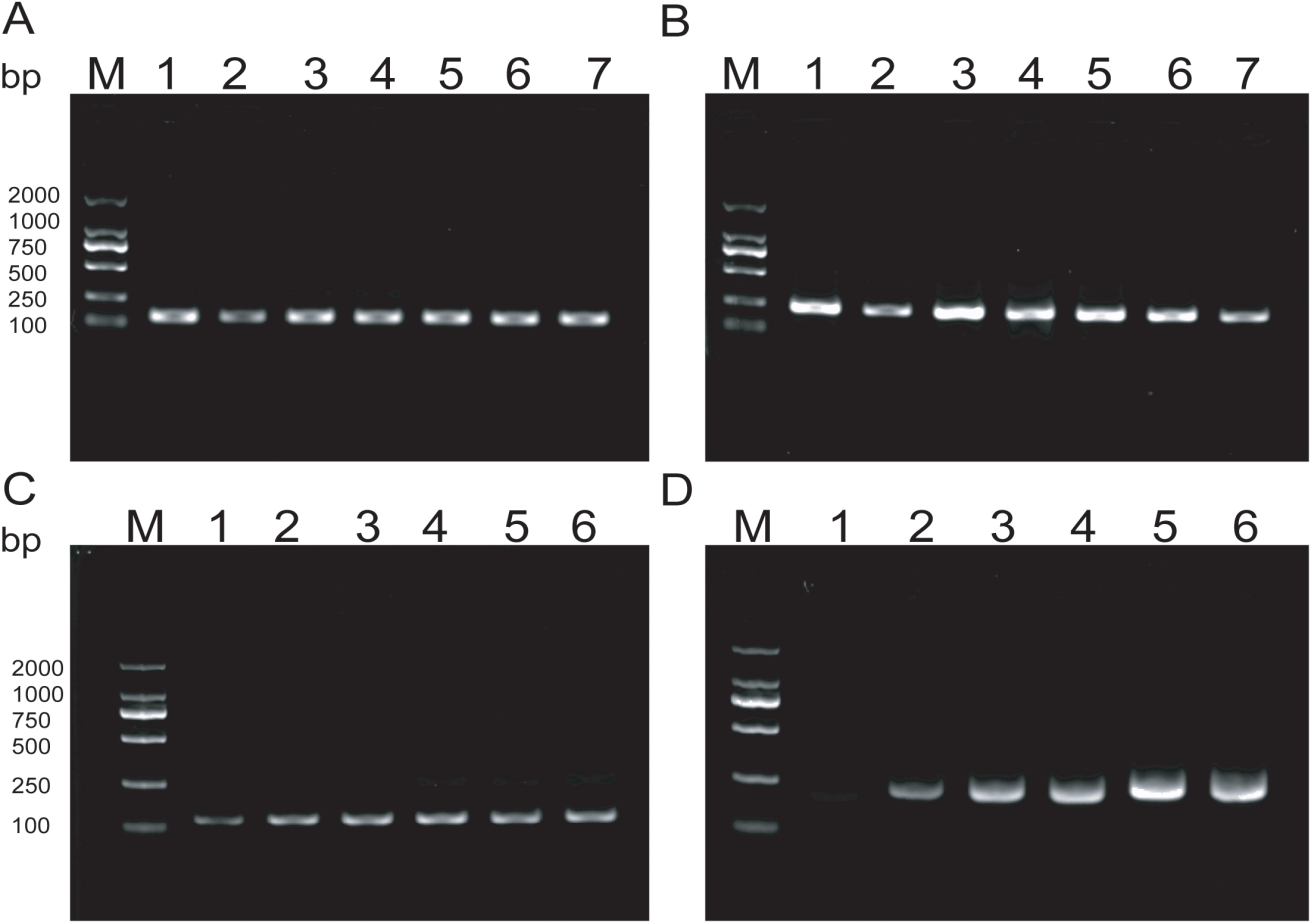
**(A, B)** Optimization of RAA reaction temperature ranging from 36°C to 42°C; **(C, D)** Optimization of RAA reaction time, The reaction time is respectively 5min, 10min, 15min, 20min, 25min, 30min.

Amplification products were detectable at reaction temperatures ranging from 36°C to 42°C. The amplification efficiency for MTB remained relatively consistent across these temperatures, whereas MAC exhibited the highest amplification efficiency at 38°C. Both exhibited consistent amplification efficiency at 37°C (**Figures 3A, B**). To ensure the stability of amplification and consistency of amplification efficiency in the duplex system, the optimal reaction temperature was ultimately determined to be 37°C. For MTB, the target band was visible at 5min into the reaction and became progressively clearer with longer incubation times (**Figure 3C**). In contrast, the band for MAC was more pronounced at 25min (**Figure 3D**). Based on these observations, the optimal duration for the duplex RAA assay was established as 20min.

### 3.2 Analytical sensitivity

**Figure 4:**
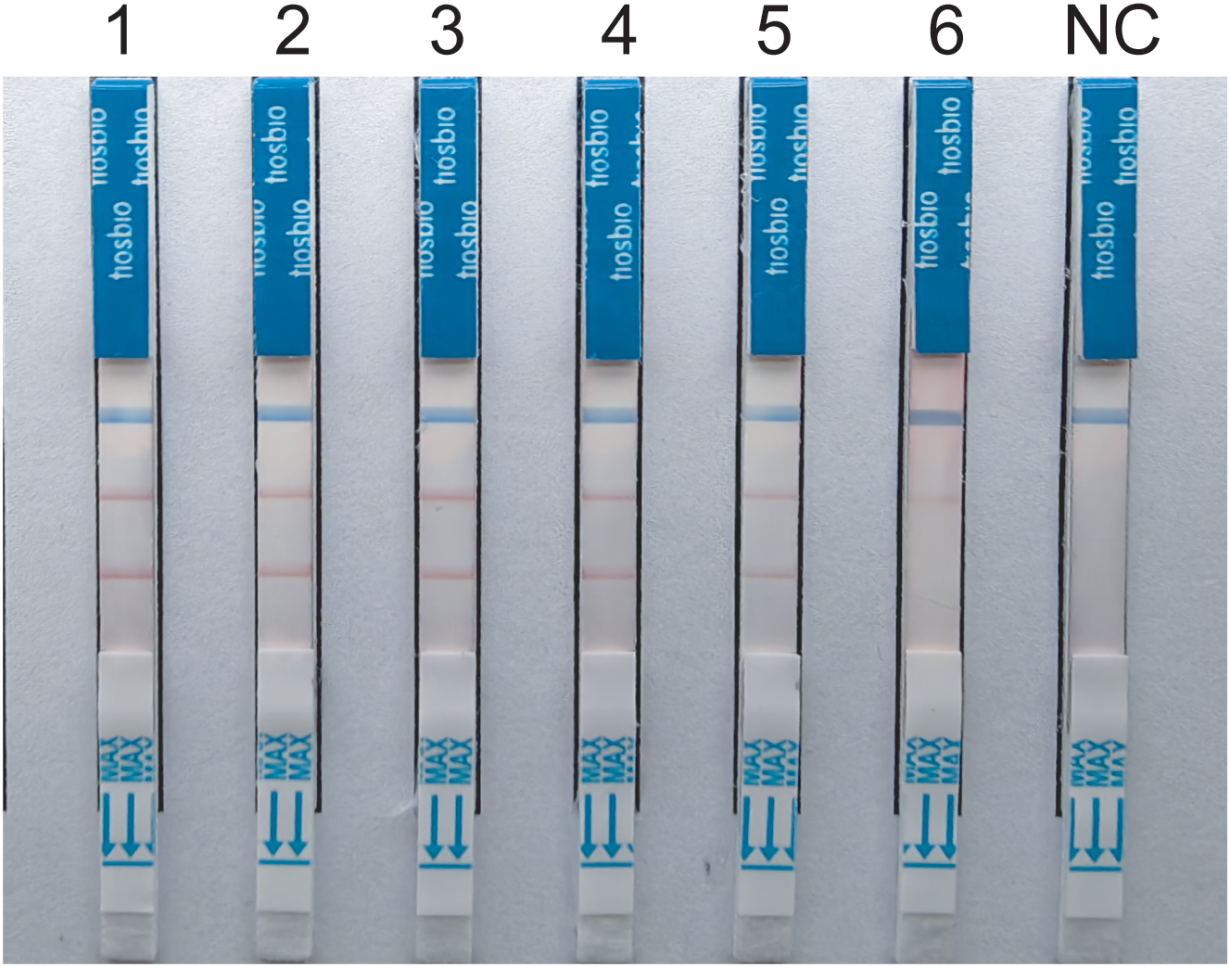
Sensitivity tests for duplex LFD-RAA assay.1-6, correspond to 10^7^-10^2^ CFU/mL and negative control, respectively.

The experimental results indicate that the lower limit of detection sensitivity for the duplex LFD-RAA assay can reach 10^2^CFU/mL. Specifically, the detection limit is 6.2×10^3^CFU/mL for MTB, and the detection limit is 8.4×10^2^CFU/mL for MAC, respectively (**Figure 4**).

### 3.3 Analytical specificity

**Figure 5:**
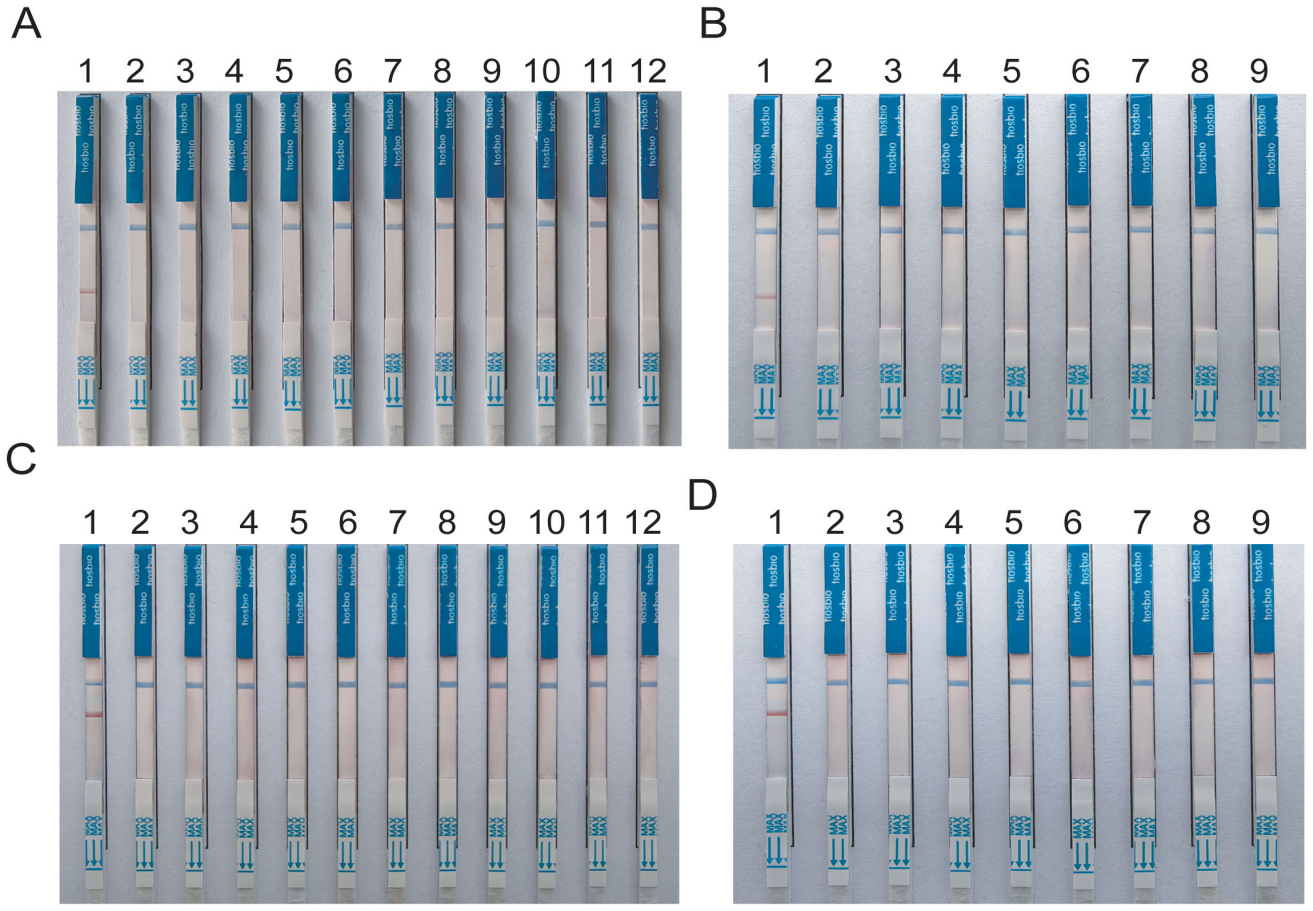
Specificity tests for detect MTB and MAC **(A, B)** DNA templates corresponding to MTB *M.kansassi*, *M.intracellulare*, *M.avium*, *M.abscessus*, *M.gordonae*, *M.chelonae*, *M.fortuitum*, *M.scrofulaceum*, *M.malmoense*, *M.chimaera*, *Klebsiella pneumoniae*, *Pseudomonas aeruginosa*, *Streptococcus pneumoniae*, *Hemophilus influenzae*, *Acinetobacter baumannii*, *Staphylococcus aureus*, *Stenotrophomonas maltophilia*. **(C, D)** DNA templates corresponding to *M.intracellulare*, MTB, *M.szulgai*. The negative control was sterile water.

Specificity analysis revealed that only the positive samples showed a distinct red band on the colloidal gold test strips, while other NTM, common respiratory bacteria, and the blank control only showed a single blue control line (**Figure 5**). This indicates that the primer-probe set in the RAA-LFD detection amplification demonstrated good specificity, effectively detecting MTB and MAC without cross-reacting with other pathogens.

### 3.4 Analytical repeatability

Inter-batch repeatability experiments were performed using two different concentrations with three replicates each. The results showed that the gray values of the detection lines for MTB at medium and low concentrations were 172.43 ± 3.725 and 156.93 ± 3.22, with CV of 2.16% and 2.05%, respectively. For MAC, the gray values at medium and low concentrations were 164.45 ± 5.10 and 137.78 ± 3.03, with CV of 3.10% and 2.20%, respectively. These results suggested that the duplex RAA-LFD demonstrated excellent repeatability and reproducibility.

### 3.5 Application of the RAA-LFD assay in clinical samples

To further evaluate the clinical performance, a total of 60 clinical samples (49 BALF and 11 sputum) were subjected to the RAA assay, acid-fast bacilli smear microscopy, liquid culture method, LAMP as well as Xpert/MTB RIF. Using Xpert as gold standard, the sensitivity of RAA-LFD assay is 92.86%, which was significantly higher than 50.0% by LAMP (**Table 2**). Among the 60 subjects, the accuracy of the duplex RAA-LFD method (76.67%) was higher than that of the acid-fast bacilli smear microscopy (51.67%) and the liquid culture method (71.67%)(**Table 3**).When clinical diagnosis was used as the standard, the sensitivity of the duplex RAA-LFD method (67.50%) was better than that of the AFB smear microscopy (27.50%) and the liquid culture method (57.50%), with the difference being statistically significant (χ^2^ = 18.65, *p*<0.001; χ^2^ = 20.93, *p*<0.005) (**Table 3**). Among the 49 patients who tested negative for AFB smear microscopy, the positive detection rates of the liquid culture method and the duplex RAA-LFD method were 24.49% (12/49) and 34.69% (17/49), respectively, with the duplex RAA-LFD method showing a significantly higher detection rate than the liquid culture method (χ^2^ = 10.96, *p*<0.001). Based on clinical diagnosis, the sensitivity of the duplex RAA-LFD method was 55.17%, compared to 41.30% for the liquid culture method, indicating the duplex RAA-LFD method had more prominent sensitivity than the liquid culture method (χ^2^ = 13.15, *p*<0.001) (**Table 4**). It is particularly noteworthy that among the 17 patients with confirmed *mycobacterial* infection, where both smear and culture results were negative, the duplex RAA-LFD was able to additionally identify 5 cases (29.4%), significantly improving the diagnostic rate for *mycobacterial* infection.

**Table 2:** Prediction value of mycobacteria infection using duplex RAA-LFD assay based on Xpert/MTB RIF.

Detection method		Xpert/MTB RIF/n		sensitivity %	specificity %	Positive predictive value %	Negative predictive value %	*Kappa*	*p*
		+(n=40)	-(n=20)						
LAMP	+	14	2	50.00	93.75	87.50	68.18	0.45	<0.001
	–	14	30						
duplex RAA-LFD	+	26	2	92.86	93.75	92.86	93.75	0.87	<0.001
	–	2	30						

**Table 3:** Prediction value of mycobacteria infection using duplex RAA-LFD assay based on clinical diagnosis.

Detection method		Clinical diagnosis/n		sensitivity (%)	specificity(%)	Positive predictive value (%)	Negative predictive value (%)	Accuracy(%)	Kappa	Standard error	*p*
		+(n=40)	-(n=20)								
AFB	+	11	0	27.50	100.00	100.00	40.82	51.67	0.20	0.064	0.009
	–	29	20								
liquid culture	+	23	0	57.50	100.00	100.00	54.05	71.67	0.47	0.092	<0.001
	–	17	20								
Xpert/MTB RIF	+	28	0	70.00	100.00	100.00	62.50	80.00	0.61	0.093	0.001
	–	12	20								
LAMP	+	16	0	40.00	100.00	100.00	45.45	60.00	0.31	0.097	<0.001
	–	24	20								
duplex RAA-LFD	+	27	1	67.50	95.00	96.43	59.83	76.67	0.54	0.098	<0.005
	–	13	19								

**Table 4:** Comparison of the efficacy of liquid culture and duplex RAA-LFD in the detection of mycobacteria in patients with negative AFB.

Detection method		Clinical diagnosis/n		sensitivity %	specificity %	Positive predictive value %	Negative predictive value %	*Kappa*	*p*
		+(n=29)	-(n=20)						
liquid culture	+	12	0	41.30	100.00	100.00	54.05	0.37	<0.001
	–	17	20						
duplex RAA-LFD	+	16	1	55.17	95.00	94.12	59.38	0.46	0.002
	–	13	19						

## 4 Discussion

The epidemiology of TB and NTM infections underscores the substantial global health impact of *mycobacterial* diseases, with significant challenges in accurate and timely diagnosis (Kumar and Kon, 2021). Although advances in molecular diagnostics have improved the detection and differentiation of TB and NTM infection, limitations in sensitivity, specificity, availability, and cost remain significant barriers (Sali et al., 2016). Addressing these challenges requires ongoing research and development of new diagnostic technologies, alongside efforts to improve the availability and affordability of existing methods in low-resource settings (Liu et al., 2022). Acid-fast Bacilli (AFB) Smear Microscopy which is rapid, inexpensive method lacks sensitivity, especially in patients with HIV co-infection or extrapulmonary TB (Dinic et al., 2013). Considered the gold standard, culture methods are more sensitive but require weeks for results, delaying diagnosis and treatment (Karuniawati et al., 2022). NAATs offer rapid, sensitive, and specific detection of MTB (Feng et al., 2022). However, NAATs require technical expertise and are more expensive, limiting their use in resource-limited settings (Malhotra et al., 2023). The application of clinical mNGS for diagnosing respiratory infections improves etiology diagnosis, but it has not been popularized in the laboratory because of its high cost (Miao et al., 2022).

Isothermal amplification technologies are designed to amplify nucleic acids at a constant temperature, eliminating the need for the thermal cycling required in PCR (Boonbanjong et al., 2022; Zhao et al., 2015). This feature simplifies the instrumentation required for nucleic acid amplification, making these technologies more accessible for POCT and in resource-limited settings (Craw and Balachandran, 2012). Various isothermal amplification methods have been developed, including Loop-mediated Isothermal Amplification (LAMP), Nucleic Acid Sequence Based Amplification (NASBA), Helicase-Dependent Amplification (HDA), and Recombinase Polymerase Amplification (RPA) (Wong et al., 2018; Jeong et al., 2009; Munawar, 2022). Recent advancements in RAA technology have focused on enhancing its specificity and sensitivity (Tian et al., 2023). Modifications to primer designs and the introduction of novel recombinase and polymerase enzymes have significantly reduced non-specific amplification and increased the limit of detection to a few molecules of target DNA. One of the most significant innovations in RAA technology is its integration with CRISPR-Cas systems, specifically Cas12 and Cas13, for ultra-sensitive and specific detection of nucleic acids (Li et al., 2023). This combination, known as CRISPR-RAA, allows for the detection of specific DNA or RNA sequences with high precision, leveraging the collateral cleavage activity of Cas proteins to signal the presence of the target nucleic acid. Advancements in RAA technology have led to the development of portable, user-friendly devices for POCT (Zhang et al., 2021). These devices often integrate RAA with lateral flow assays or fluorescence detection, providing rapid results without the need for sophisticated laboratory equipment (Liu et al., 2023; Xia et al., 2022). Recent studies have demonstrated the potential for duplexing RAA reactions, allowing for the simultaneous detection of multiple targets in a single assay (Cao and Song, 2023; Ma et al., 2023). This is particularly useful for differential diagnosis of diseases with overlapping symptoms or for detecting co-infections. RAA offers many advantages over traditional PCR, however, challenges remain in its widespread adoption. The optimization of reaction conditions for different targets, the potential for carry-over contamination, and the need for further validation in clinical settings are areas that require ongoing research (Munawar, 2022). Future directions for RAA technology include the development of more robust reaction mixes that are tolerant of inhibitory substances commonly found in clinical samples, enhancing the utility of RAA in a wider range of applications.

The RAA-assisted isothermal amplification technique developed in this study represents a significant advancement in the field of TB diagnostics. To our knowledge, we develop a novel RAA based molecular assay for rapid amplification of MTB and MAC from clinical specimens. Our findings demonstrate that the method shows high specificity and sensitivity for detecting MTB and differentiating it from MAC, and the whole amplification process takes only 20min. This performance surpasses that of traditional diagnostic methods, addressing critical gaps in the timely and accurate diagnosis of TB and MAC infections. The clinical applicability of our RAA-based method, particularly in resource-limited settings, is among its most significant advantages. In this context, there is an urgent need for simple, rapid, and accurate diagnostic tools, as delayed or incorrect diagnoses can lead to ongoing transmission and inappropriate treatment regimens, thereby exacerbating the public health burden of *mycobacterial* diseases. Furthermore, our study highlights the importance of integrating molecular diagnostics with clinical and epidemiological data to enhance the management of TB and MAC infections. The ability to rapidly distinguish between these infections enables targeted therapeutic interventions, reducing the risk of drug resistance and improving therapeutic effect.

However, challenges remain in the broader implementation of RAA-based diagnostics, including the need for further validation in diverse settings and among varied patient populations. Future research should focus on operationalizing this technology in field conditions, evaluating its performance against a broader array of *mycobacterial* species, and assessing its cost-effectiveness compared to existing diagnostics. We compared five methods for detecting *mycobacteria*, including duplex RAA-LFD. The results showed that the positive detection rate, sensitivity and specificity of duplex RAA-LFD in sputum samples of pulmonary TB patients were higher than that of AFB and liquid culture. More importantly, the duplex RAA method has high diagnostic efficacy in both TB infection and smear-negative TB, and has high application value for the early and rapid diagnosis of clinical *mycobacterium* infection and MAC differential diagnosis, especially for those with negative smear tests. Besides, it has the characteristics of short time, simple operation and low detection cost, which is worthy of further clinical verification.

So far most isothermal nucleic acid amplification techniques are usually limited to amplify only one target sequence (Mayboroda et al., 2018). First of all, the entire reaction of RAA is performed by quantitative enzyme system instead of ordinary PCR through the process of unchain amplification through variable temperature, and the multiple systems lead to competitive inhibition due to the consumption of enzymes (Piepenburg et al., 2006). Secondly, due to the restriction of the band size of the amplification target, usually RAA nucleic acid products are between 125-200bp, and it is difficult to distinguish them by agarose detection (Tan et al., 2022). In our study, we found that by continuously optimizing the concentration of probe primers in the reaction system, the efficiency of the two groups of amplification systems was similar. Further, different antigen-modified primers and probes were used for visual differential diagnosis of LFD.

In conclusion, the RAA-assisted isothermal amplification method offers a promising approach for the rapid, specific, and sensitive discrimination of TB from MAC. In future research, we are continuously developing RAA-based diagnostics for other NTM species, such as *Mycobacterium kansasii* and *Mycobacterium abscessus*, with the goal of achieving rapid diagnosis coverage for 99% of mycobacteria. We will continue refining the multiplex system to enable simultaneous detection of multiple pathogens and facilitate further clinical utility. Its implementation could significantly improve the diagnosis and management of mycobacterial diseases, particularly in settings where traditional diagnostic resources are limited. Continued innovation and research in this area are essential for advancing global TB control and prevention efforts.

## Data Availability

The raw data supporting the conclusions of this article will be made available by the authors, without undue reservation.
